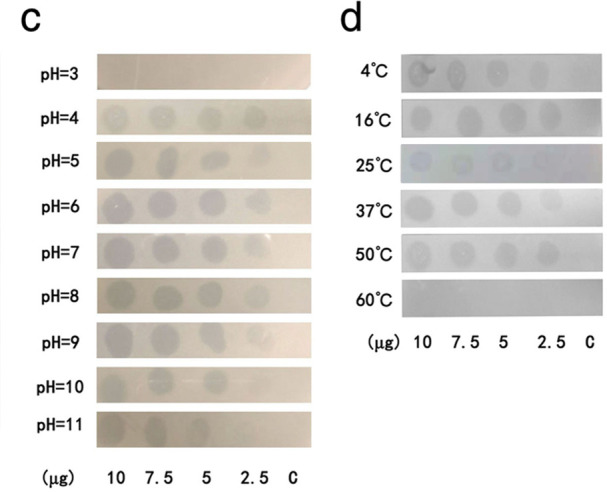# Correction for Zhao et al., “A novel phage putative depolymerase, Depo16, has specific activity against K1 capsular-type *Klebsiella pneumoniae*”

**DOI:** 10.1128/aem.02170-25

**Published:** 2025-12-08

**Authors:** Rihong Zhao, Shanshan Jiang, Siyu Ren, Li Yang, Wenyu Han, Zhimin Guo, Jingmin Gu

## AUTHOR CORRECTION

Volume 90, no. 4, e01197-23, 2024, https://journals.asm.org/doi/10.1128/aem.01197-23. Page 9: Fig. 3c and d should appear as shown in this correction. We inadvertently used the wrong image during the drawing process. However, the conclusion remains valid. We apologize for this error, which did not change the final result.

**Fig 3:**